# Increasing Prevalence of Multidrug-Resistant *Candida haemulonii* Species Complex among All Yeast Cultures Collected by a Reference Laboratory over the Past 11 Years

**DOI:** 10.3390/jof6030110

**Published:** 2020-07-15

**Authors:** Soraia Lopes Lima, Elaine Cristina Francisco, João Nóbrega de Almeida Júnior, Daniel Wagner de Castro Lima Santos, Fabiane Carlesse, Flávio Queiroz-Telles, Analy Salles de Azevedo Melo, Arnaldo Lopes Colombo

**Affiliations:** 1 Division of Infectious Diseases, Escola Paulista de Medicina-Universidade Federal de São Paulo, São Paulo 04024-002, Brazil; soraia_lps@hotmail.com (S.L.L.); elaineperol@yahoo.com.br (E.C.F.); danielinfectologista@gmail.com (D.W.d.C.L.S.); analysalles@gmail.com (A.S.d.A.M.); 2 Divisão de Laboratório Central-LIM03, Hospital das Clínicas da FMUSP, São Paulo 01246-100, Brazil; jnaj99@gmail.com; 3 Instituto de Oncologia Pediátrica, Universidade Federal de São Paulo, São Paulo 04023-062, Brazil; fabiannecarlesse@graacc.org.br; 4 Departamento de Saúde Pública, Universidade Federal do Paraná, Curitiba 80.060-240, Brazil; queiroz.telles@uol.com.br

**Keywords:** *Candida haemulonii* species complex, cryptic *Candida* species, candidemia, antifungal resistance, nosocomial fungal infections

## Abstract

There is worldwide concern with the increasing rates of infections due to multiresistant *Candida* isolates reported in tertiary medical centers. We checked for historical trends in terms of prevalence rates and antifungal susceptibility of the *Candida haemulonii* species complex in our yeast stock culture collected during the last 11 years. The isolates were identified by sequencing the rDNA internal transcribed spacer (ITS) region, and antifungal susceptibility tests for amphotericin B, voriconazole, fluconazole, anidulafungin, and 5-fluorocytosine were performed by the Clinical and Laboratory Standards Institute (CLSI) microbroth method. A total of 49 isolates were identified as *Candida haemulonii* *sensu stricto* (*n* = 21), followed by *C. haemulonii* var. *vulnera* (*n* = 15) and *C. duobushaemulonii* (*n* = 13), including 38 isolates cultured from patients with deep-seated *Candida* infections. The prevalence of the *C. haemulonii* species complex increased from 0.9% (18 isolates among 1931) in the first period (December 2008 to June 2013) to 1.7% (31 isolates among 1868) in the second period (July 2014 to December 2019) of analysis (*p* = 0.047). All isolates tested exhibited high minimum inhibition concentrations for amphotericin B and fluconazole, but they remained susceptible to 5-fluorocytosine and anidulafungin. We were able to demonstrate the increased isolation of the multiresistant *Candida haemulonii* species complex in our culture collection, where most isolates were cultured from patients with deep-seated infections.

## 1. Introduction

*The Candida haemulonii* species complex comprises emerging opportunistic yeast pathogens represented by *C. haemulonii sensu stricto, C. haemulonii var. vulnera, and C. duobushaemulonii* that are considered to be closely related to *C. pseudohaemulonii* and *C. auris* [1]. This species complex is able to cause superficial and deep-seated infections, including candidemia, especially in neonates, patients with cancer and/or diabetes mellitus, as well as critically ill patients exposed to invasive medical procedures and antibiotics [2,3,4].

The prevalence of *C. haemulonii* and *C. duobushaemulonii* in medical centers that use classical phenotypic identification methods (e.g., Vitek 2, Biomérieux) may be underestimated since these systems cannot accurately ascertain these species [1,2,3,4,5]. Accurate identification of this species complex has been carried out by molecular tools or by matrix-assisted laser desorption ionization time-of-flight mass spectrometry (MALDI-ToF MS) [1,5,6], and this represents a crucial step in driving antifungal therapy once these pathogens can be frequently characterized as multidrug resistant (MDR), exhibiting a high minimum inhibitory concentration (MIC) to amphotericin B (AMB) and triazoles [7,8,9,10].

The majority of publications addressing the *C. haemulonii* species complex are based on single-center experiences and small series [3,7,10], with the exception of three multicenter studies published in the last years that analyzed 30, 31, and 40 isolates each [1,4,11]. In order to check for historical trends in the prevalence rate of species and their antifungal susceptibility results, we checked for all *C. haemulonii* species complex isolates in our yeast culture collection obtained during the last 11 years, including samples collected from patients admitted in 12 medical centers.

## 2. Materials and Methods

### 2.1. Selection of C. haemulonii Species Complex Clinical Isolates

We selected a total of 49 clinical isolates of the *C. haemulonii* species complex that had been stored in the yeast culture collection at Laboratório Especial de Micologia, Escola Paulista de Medicina, Universidade Federal de São Paulo, São Paulo, Brazil. All clinical isolates were sent to our center by routine laboratories from 12 hospitals in Brazil including all sequential isolates that were cultured from patients assisted during the period between 2008 and 2019. The yeasts were sequentially cultured from different patients (49), including samples from diverse anatomic sites. In order to check for the prevalence rates of species within the *C. haemulonii* species complex in our yeast culture collection, we included, as denominator, a total of 3799 yeast isolates that were stored in our laboratory along the same period of time. Sequential isolates obtained from the same patients were excluded from the analysis. This study was approved by the ethics committee of our university (CEP-UNIFESP Number 5454270220, approved on 16 March 2020).

### 2.2. Molecular Identification by Sequencing of rDNA ITS

The identification of isolates was carried out as previously described [12]. PCR, for amplification of the internal transcribed spacer (ITS) rDNA region, was performed using the primer pair V9G (5′-TTACGTCCCTGCCCTTTGTA-3′) and LS266 (5′-GCATTCCCAAACAACTCGACTC-3′) [13,14]. Sequencing of the amplicons was performed using the forward primers V9G and ITS1 (5′-TCCGTAGGTGAACCTGCGG-3′) and the reverse primers LS266 and ITS4 (5′-TCCTCCGCTTATTGATATGC-3′) [14,15]. Sequences were assembled based on four reads per isolate and edited in Sequencher 4.1.4 (Gene Codes Corporation, Ann Arbor, MI, USA). The ITS sequences were aligned and edited using the muscle algorithm implemented in SeView software 4.2.12 [16] excluding 18S and 28S rDNA.

The consensus was compared to reference sequences deposited in the GenBank and ISHAM Barcoding Databases (https://blast.ncbi.nlm.nih.gov; http://its.mycologylab.org). Accurate species identification targets included an *E*-value of ≤10^−5^ and identity and coverage of ≥98% [12,13].

The final identification at species level was confirmed by phylogenetic analysis using the neighbor-joining method based on the Kimura two-parameter model with 1000 bootstrap pseudo-replicates, including type sequences of the *C. haemulonii* species complex in SeaView [17].

The rDNA ITS sequences of *Candida haemulonii* species complex clinical strains were deposited in the GenBank database. For complete information, including strain number, species name, and accession numbers, see Table S1.

### 2.3. Antifungal Susceptibility Tests

Antifungal susceptibility tests for AMB (Sigma Aldrich, St. Louis, MI, USA), voriconazole (VRC, Sigma), fluconazole (FLC, Sigma), anidulafungin (AFG, Sigma), and 5-fluorocytosine (5FC, Sigma) were performed using the CLSI broth microdilution method [18]. The concentrations tested ranged between 0.03 and 16 µg/mL for all drugs, except FLC (ranged from 0.125 to 64 µg/mL). MIC values were determined after 48 h of incubation as suggested by Cendejas-Bueno et al. [1].

### 2.4. Statistical Analysis

In order to check for historical trends in species distribution, prevalence rates of the *C. haemulonii* species complex were checked taking into consideration all yeast samples collected along two different time periods: (Ι) period 1 (P1) with 1931 yeast isolates (including 18 *C. haemulonii* species complex) collected between December 2008 and June 2013; (ΙΙ) period 2 (P2) with 1868 yeast isolates (including 31 *C. haemulonii* species complex) collected between July 2014 and December 2019. Prevalence rates in both periods (P1 vs. P2) were compared by chi-square tests. Finally, antifungal MIC values obtained for each of the three *C. haemulonii* cryptic species were compared by a Kruskal–Wallis test. All tests with *p* < 0.05 were considered statistically significant.

## 3. Results

### 3.1. Prevalence Rates of the C. haemulonii Species Complex

The 49 yeast isolates were cultured from the following clinical samples: blood/central venous catheter tips (*n* = 33; 67.5%), tissue biopsies (*n* = 4; 8%), respiratory tract fluids (*n* = 4; 8%), skin/vaginal samples (*n* = 6; 12.5%), spinal fluid (*n* = 1; 2%), and urine (*n* = 1; 2%). The 38 isolates (77.5%), collected from blood/catheter tips, tissue biopsies, and spinal fluid, were implicated in causing episodes of deep-seated *Candida* infections, in contrast with the 11 (22.4%) isolates recovered from the skin, vagina, urine, and respiratory tract that were considered as colonizers.

Taking all 49 isolates tested, *C*. *haemulonii sensu stricto* was the most common species found (*n* = 21; 43%), followed by *C. haemulonii* var. *vulnera* (*n* = 15; 30.5%) and *C. duobushaemulonii* (*n* = 13; 26.5%). Considering only the 38 isolates related to episodes of deep-seated *Candida* infections, *C. haemulonii sensu stricto* remained the most common species found (42%), followed by *C. haemulonii* var. *vulnera* and *C. duobushaemulonii*. Notably, *C*. *haemulonii sensu stricto* was present in five (45%) out 11 isolates considered as colonizers.

**Figure 1:**
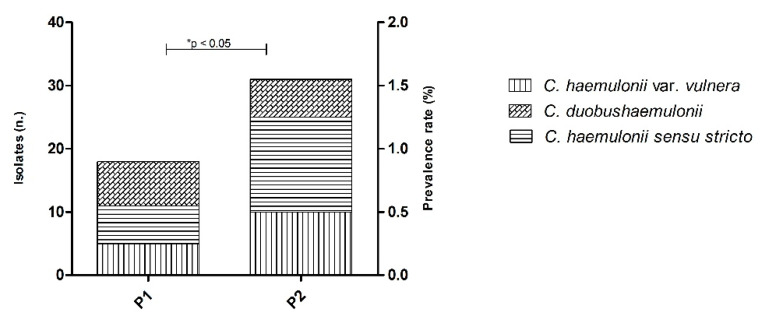
Prevalence rates of *C. haemulonii* species complex clinical isolates. P1: December 2008 to June 2013; P2: July 2013 to December 2019.

In terms of historical trends in species distribution documented in our yeast stock collection during the last 11 years, we noted that the prevalence of *Candida haemulonii* species complex isolates increased from 0.9% (18 isolates among *n* = 1931) in the first period to 1.7% (31 isolates among *n* = 1868) in the second period of analysis (*p* = 0.047) (Figure 1). No substantial changes in the frequency of the isolation of species within the *C. haemulonii* species complex were documented in both periods, and *C. haemulonii sensu stricto* remained as the most common species found during the 11 years (P1 *n* = 6; 33.4% vs. P2 *n* = 15; 48.3%, *p* > 0.05).

### 3.2. Antifungal Susceptibility Profiles

As summarized in Table 1, the MIC values for the 49 clinical isolates of the *C. haemulonii* species complex ranged from 1 to ≥16 µg/mL for AMB, 1 to ≥64 µg/mL for FLC, 0.03 to ≥16 µg/mL for VRC, 0.06 to 1 µg/mL for 5FC, and 0.03 to 0.5 µg/mL for AFG.

We were able to find some species-specific patterns of antifungal susceptibility in our collection, where *C. duobushaemulonii* isolates exhibited the highest AMB MIC values (GM = 3.41 μg/mL) when compared to *C. haemulonii sensu stricto* (GM = 1.64 μg/mL) and *C. haemulonii* var. *vulnera* (GM = 1.74 μg/mL; *p* < 0.05). In contrast, *C. duobushaemulonii* isolates presented lower VRC MIC values (GM = 0.36 μg/mL) when compared with MIC results obtained from isolates of the other two species tested (GM = 1.03 and 1.44 μg/mL, *p* < 0.05). Finally, regardless of the species considered, all isolates tested exhibited high FLC MICs GMss ranging from 10.42 to 17.80), but remained highly susceptible to 5FC and AFG (GMs ranging from 0.10 to 0.20 µg/mL).

Regarding the antifungal susceptibility tests performed with isolates cultured from different anatomical sites, we were not able to identify any significant differences between MICs generated with isolates from episodes of deep-seated infections when compared to those obtained by patients probably colonized by these pathogens.

## 4. Discussion

Despite the *C*. *haemulonii* species complex being considered among the rare human pathogens, concerns about their incidence rates and antifungal resistance appear to be increasing worldwide [1,3,11]. In the present study, we found a prevalence rate of 1.3% of the *C. haemulonii* species complex in our Brazilian yeast collection of 3799 clinical isolates stored over the last 11 years. Other authors have reported prevalence rates of the *C. haemulonii* species complex ranging between 0.01% and 0.3% of their yeast stock cultures [4,11,19,20].

Remarkably, we were able to demonstrate a substantial increase in the isolation of *C. haemulonii* species complex during the last 11 years, from 18 out 1931 (0.9%) isolates cultured in P1 versus 31 out 1868 (1.7%) isolates identified in P2 (*p* = 0.047). The facts behind the emergence of this species complex are still not completely understood, but it may be related to the increasing rates of patients submitted to invasive medical procedures, the selective pressure of prophylaxis, and empirical therapy with antifungals [1,4,21].

As reported by other authors, *C. haemulonii sensu stricto* was the most prevalent species found followed by *C. haemulonii* var. *vulnera* and *C. duobushaemulonii* when checking isolates obtained either from yeasts cultured from invasive episodes of infection or from colonized patients [1,4,11,22,23]. Otherwise, it is important to highlight that *C. haemulonii* var. *vulnera* and *C. duobushaemulonii* together were found in 57% (*n* = 28) of all isolates in our analysis.

In terms of their antifungal susceptibility, all *C. haemulonii* species complex isolates we tested exhibited high MIC values against AMB and FLC, a finding that was already reported by several investigators [7,8,9,10,11]. In contrast, they all exhibited low MIC_50_ and MIC_90_ values against 5FC and anidulafungin. The clinical relevance of this finding remains unclear, but most authors suggest that episodes of infections with *C. haemulonii* species complex should be treated with echinocandins, instead of using fluconazole and amphotericin B [4,7,11].

In regard to antifungal MICs obtained with isolates representative of different species within the *C. haemulonii* species complex, we found that *C. duobushaemulonii* presented higher AMB MIC values and lower results of VRC MICs when compared to the other species tested. This finding is in accordance with previous results reported by Kumar et al. [3] and Cendejas-bueno et al. [1].

The molecular mechanisms involved in antifungal resistance with *C. haemulonii* species complex isolates are not completely understood. However, initial data suggest that substitutions in the *ERG11* gene, chromosomal duplications, and efflux pumps were found to be associated with azole resistance in Latin American *C. haemulonii* and *C. duobushaemulonii* isolates [23,24].

Finally, we were not able to identify any significant differences between MIC results generated by yeast isolates cultured from episodes of deep-seated infections when compared to those obtained from colonized patients. Notably, the limited number of isolates tested impairs any definitive conclusion in this regard.

In conclusion, our main findings were as follows: (Ι) prevalence rates of *C. haemulonii* species complex isolates increased in our yeast culture collection during the last 11 years; (ΙΙ) despite *C. haemulonii sensu stricto* representing the most common species found, 57% of isolates were identified as *C. haemulonii* var. *vulnera* or *C. duobushaemulonii*, including cultures obtained from patients with deep-seated infections; (ΙΙΙ) all species tested exhibited high MIC values against amphotericin B and fluconazole, making it clear that these fungal pathogens appear to be multiresistant, but still susceptible to echinocandins.

## Figures and Tables

**Table 1:** Antifungal susceptibility testing of 49 *C. haemulonii* species complex clinical isolates.

Species (*n*)	MICs	Antifungals Tested (µg/mL)				
		AMB	FLC	VRC	5FC	AFG
*C. haemulonii sensu stricto* (21)	MIC_50_	2	8	0.5	0.125	0.125
	MIC_90_	2	>64	>16	0.125	0.25
	GM	1.64	10.42	1.03	0.13	0.10
	Range	1–8	2–(>64)	0.03–(>16)	0.06–0.25	0.06–0.25
*C. haemulonii* var. *vulnera* (15)	MIC_50_	2	16	0.5	0.125	0.125
	MIC_90_	2	>64	>16	0.5	0.25
	GM	1.74	13.93	1.44	0.20	0.13
	Range	1–4	1–(>64)	0.03–(>16)	0.125–1	0.03–0.25
*C. duobushaemulonii* (13)	MIC_50_	4	16	0.25	0.25	0.125
	MIC_90_	>16	32	1	0.25	0.25
	GM	3.41	17.80	0.36	0.20	0.10
	Range	1–(>16)	8–(>64)	0.06–(>16)	0.125–0.5	0.03–0.5

Amphotericin B (AMB); voriconazole (VRC); fluconazole (FLC); anidulafungin (AFG); 5-fluorocytosine (5FC); minimum inhibitory concentration able to inhibit 50% of all isolates tested (MIC_50_); minimum inhibitory concentration able to inhibit 90% of all isolates tested (MIC_90_); geometric mean (GM); number of isolates tested (*n*).

## Supplementary Materials

The following are available online at https://www.mdpi.com/2309-608X/6/3/110/s1, Table S1: Ribosomal DNA ITS sequences of 49 *Candida haemulonii* species complex deposited in public sequence database. Species names, strains, and GenBank accession numbers.
