# Supplementary material for: Increasing Prevalence of Multidrug-Resistant Candida haemulonii Species Complex among All Yeast Cultures Collected by a Reference Laboratory over the Past 11 Years
Source: J Fungi (Basel). 2020 Jul 15;6(3):110. doi: 10.3390/jof6030110 (PMC7558365; doi:10.3390/jof6030110)
Supplement: Supplementary file 1 [file jof-06-00110-s001.pdf]

Isolate	Specie identification	Isolate Souce	GenBank acession number
3885	C. haemulonii sensu stricto	Blood	MH118007
546/2015	C. duobushaemulonii	Vaginal samples	MH118000
547/2015	C. duobushaemulonii	Vaginal samples	MH117999
5075	C. duobushaemulonii	Blood	KC408994
1894/2017	C. duobushaemulonii	Blood	MN172351
6066	C. haemulonii var. vulnera	Blood	MH118015
5800	C. haemulonii sensu stricto	Blood	MH118011
6083	C. haemulonii var. vulnera	Blood	MH118016
6919	C. duobushaemulonii	Blood	MH117997
582/2015	C. haemulonii var. vulnera	Blood	MH118012
583/2015	C. haemulonii var. vulnera	Blood	MH118013
8229	C. duobushaemulonii	Blood	MH117998
584/2015	C. haemulonii var. vulnera	Blood	MH118014
9124	C. duobushaemulonii	Blood	MH118003
767/2015	C. haemulonii sensu stricto	Catheter tips	MH118008
768/2015	C. duobushaemulonii	Respiratory tract fluid	MH118001
585/2015	C. haemulonii sensu stricto	Blood	MH118006
1957/2017	C. haemulonii sensu stricto	Blood	MH118009
43/2018	C. haemulonii sensu stricto	Blood	MH118010
9700A	C. haemulonii sensu stricto	Blood	MH118004
10194A	C. haemulonii var. vulnera	Tissue biopsis	MH118019
9873	C. haemulonii var. vulnera	Blood	MH118017
9822	C. haemulonii var. vulnera	Spinal fuid	MH118018
1112/2016	C. haemulonii var. vulnera	Blood	MH118021
1205/2016	C. duobushaemulonii	Catheter tips	MH118002
032/2019	C. duobushaemulonii	Blood	MT533272
135/2019	C. duobushaemulonii	Catheter tips	MT533273
210/2019	C. duobushaemulonii	Blood	MT533274
455/2019	C. duobushaemulonii	Tissue biopsis	
3834A	C. haemulonii sensu stricto	Blood	KC408993
1204/2016	C. haemulonii sensu stricto	Catheter tips	MN172335
1679/2017	C. haemulonii sensu stricto	Catheter tips	MN172336
1810/2017	C. haemulonii sensu stricto	Blood	MN172337
1887/2017	C. haemulonii sensu stricto	Tissue biopsis	MN172338
14/2018	C. haemulonii sensu stricto	Skin	MN172339
34/2018	C. haemulonii sensu stricto	Respiratory tract fluid	MN172340
1967/2017	C. haemulonii sensu stricto	Blood	MN172341
86/2018	C. haemulonii sensu stricto	Urine	MN172342
208/2018	C. haemulonii sensu stricto	Respiratory tract fluid	MN172343
274/2018	C. haemulonii sensu stricto	Skin	MN172344
302/2018	C. haemulonii sensu stricto	Blood	MT533267
048/2019	C. haemulonii sensu stricto	Blood	MT533268
127/2019	C. haemulonii sensu stricto	Catheter tips	MT533269
1811/2017	C. haemulonii var. vulnera	Blood/catheter tips	MN172345
1888/2017	C. haemulonii var. vulnera	Skin	MN172346
82/2018	C. haemulonii var. vulnera	Respiratory tract fluid	MN172348
104/2018	C. haemulonii var. vulnera	Skin	MN172349
144/2018	C. haemulonii var. vulnera	Tissue biopsis	MT533270
301/2018	C. haemulonii var. vulnera	Blood	MT533271
